# Serpin Family E Member 1 Tag Single-Nucleotide Polymorphisms in Patients with Diabetic Nephropathy: An Association Study and Meta-Analysis Using a Genetic Model-Free Approach

**DOI:** 10.3390/genes12121887

**Published:** 2021-11-25

**Authors:** Maria Tziastoudi, Efthimios Dardiotis, Georgios Pissas, Georgios Filippidis, Spyridon Golfinopoulos, Vasileios Siokas, Sophia V. Tachmitzi, Theodoros Eleftheriadis, Georgios M. Hadjigeorgiou, Evangelia Tsironi, Ioannis Stefanidis

**Affiliations:** 1 Department of Nephrology, Faculty of Medicine, School of Health Sciences, University of Thessaly, 41110 Larissa, Greece; gpissas@msn.com (G.P.); gfilippid@yahoo.gr (G.F.); spygolfin@yahoo.gr (S.G.); telefteriadis@med.uth.gr (T.E.); stefanid@med.uth.gr (I.S.); 2 Laboratory of Neurogenetics, Department of Neurology, University Hospital of Larissa, University of Thessaly, 41110 Larissa, Greece; edar@med.uth.gr (E.D.); bill_s1983@hotmail.com (V.S.); gmhadji@med.uth.gr (G.M.H.); 3 Department of Ophthalmology, Faculty of Medicine, School of Health Sciences, University of Thessaly, 41110 Larissa, Greece; sophitach@yahoo.gr (S.V.T.); etsiron@med.uth.gr (E.T.); 4 Department of Neurology, Medical School, University of Cyprus, Nicosia 22006, Cyprus

**Keywords:** *SERPINE1*, diabetic nephropathy, gene polymorphism, systematic review, meta-analysis

## Abstract

Background: Many lines of evidence highlight the genetic contribution on the development of diabetic nephropathy (DN). One of the studied genes is *SERPINE1* whose the role in the risk of developing DN remains questionable. In order to elucidate the contribution of *SERPINE1* in DN progression in the context of type 2 diabetes mellitus (T2DM), we conducted an association study and meta-analysis of *SERPINE1* genetic variants. Materials and Methods: A total of 190 patients with DN, 150 T2DM (type 2 diabetes mellitus) patients without DN and 238 healthy controls were recruited. We selected five tag single-nucleotide polymorphisms (SNPs) from the HapMap. The generalized odds ratio (OR_G_) was calculated to estimate the risk on DN development. Subgroup analyses based on ethnicity and type of diabetes were also performed. Results: Both the present association study regarding *SERPINE1* SNPs (rs2227667, rs2070682, rs1050813, rs2227690, rs2227692) did not found any significant association between *SERPINE1* variants and DN and the meta-analysis of variant 4G>5G (rs1799889) did not also reveal a significant association between 4G>5G variant and DN in main and subgroup analyses. Discussion: In conclusion, the present association study and meta-analysis provides strong evidence that *SERPINE1* genetic variant 4G>5G is not implicated in the risk or development of DN in Caucasians. Further studies in other populations remain to further investigate the role of this variant in the course of DN.

## 1. Background

Diabetic nephropathy (DN) constitutes one of the microvascular complications of both type 1 diabetes mellitus (T1DM) and type 2 diabetes mellitus (T2DM) and the most common form of chronic kidney disease [1]. Despite the glycemic and hemodynamic alterations, the genetic contribution in DN is unquestionable but still obscure [2,3]. Many genetic loci have been implicated in the pathogenesis of the disease but each genetic loci is characterized of small effect size [4,5].

Two main approaches were used for the genetic dissection of the DN, genetic linkage studies and genetic association studies [6,7]. There is a plethora of genetic linkage studies [8,9,10,11,12,13] and genetic association studies that examines one or more loci or even refers to genome-wide scale [14,15,16,17,18,19,20].

Except individual studies, there are also available meta-analyses of these studies. The most recent meta-analysis of genome-wide linkage studies (GWLS) identified significantly suggestive for linkage with DN cytogenetic locations on the following chromosomes: 1q, 3q, 4p, 5q, 7q, 15q, 16p, 17q, 19q and 22p [21]. On the other hand, the most recent meta-analysis of genetic association studies in DN revealed significance of genetic polymorphisms harbored in fifty-one loci and three intergenic regions [22]. In another meta-analysis of genetic association studies, the pathway analysis of significant genes revealed statistically significant overrepresentation of six signalling pathways: cytokine–cytokine receptor interaction, pyruvate metabolism, T2DM, adipocytokine signalling pathway, renal cell carcinoma and the renin–angiotensin system [23]. 

Among the numerous genetic loci studied in both individual studies [24,25,26,27,28,29,30,31] and meta-analyses [32,33,34] is serpin family E member 1 (*SERPINE1*) gene, also known as Plasminogen activator inhibitor-1 (PAI-1), which is a member of the serine proteinase inhibitor (serpin) superfamily and constitutes the principal inhibitor of tissue plasminogen activator (tPA) and urokinase (uPA) leading to inhibition of fibrinolysis [32]. The *SERPINE1* gene is located at chromosome band 7q22.1. The protein also functions as a component of innate antiviral immunity. It is known a common polymorphism in the promoter region of the gene, known as 4G/5G (rs1799889), with the 5G allele slightly less transcriptionally active than the 4G allele. Increased PAI-1 levels are associate with diabetic complications [35,36,37]. 

In this study, in an effort to enlighten the contribution of *SERPINE1* gene in the pathogenesis of DN in the context of T2DM, we selected five tag single-nucleotide polymorphisms (SNPs) for genotyping in a case-control study of Caucasians. In order to confirm the findings of the study, we also performed a meta-analysis of available genetic polymorphisms located in this gene.

## 2. Materials and Methods

### 2.1. Association Study

#### 2.1.1. Study Population

The details about the study design and the participants have been described elsewhere [38]. In brief, a total of 190 patients with DN, 150 T2DM patients without microvascular complications and 238 healthy controls were participated in this study. All participants were examined in the Ophthalmology and Nephrology outpatient clinics of the University Hospital of Larissa in Greece. 

The criteria of diabetic nephropathy (DN) was persistent albuminuria, urinary albumin excretion>300 mg/24 h (>200 μg/min) regardless the elevated or not serum creatinine levels. The study was approved by the University of Thessaly Ethics Committee and informed consent was received from all participants.

#### 2.1.2. Genotyping

Genomic DNA was extracted from peripheral blood samples using a salting out method. Based on the HapMap population database for Utah residents with Northern and Western European ancestry (CEU) (Release 27, Phase II+III, Feb09, on NCBI B36 assembly, dbSNP b126) tag single nucleotide polymorphisms (SNPs) across *SERPINE1* (spanning a 11.86 kbp region that consists of nine exons in chr7:positions 100,557,172 to 100,569,026) were identified on the basis of linkage disequilibrium (LD) blocks according to HapMap project (http://hapmap.ncbi.nlm.nih.gov accessed on 15 May 2016) using the tagger genetic program (http://www.broadinstitute.org/mpg/tagger accessed on 15 May 2016). Selection of tagging SNPs was conducted using criteria of r^2^ cut-off of greater than or equal to 0.8 and minor allele frequency (MAF) of >0.05. The details of the selection of tagging SNPs have been also described elsewhere(38). A total of 5 tag SNPs in four distinct gene regions were retrieved in the intronic region between exons 3–4 (rs2227667), in the intron 5–6 (rs2070682, rs2227690), the intron 7–8 (rs2227692), and in the 3′UTR region (rs1050813). The captured tag SNPs were distributed in two specific LD blocks: rs2227667 in block 1 and rs2070682, rs2227690, rs2227692, and rs1050813 in block 2.

Genotyping of tag SNPs was performed with a TaqMan allele specific discrimination assays method on an ABI PRISM 7900 sequence detection system and was analyzed with the SDS software version 2.3 (Applied Biosystems, Foster City, CA, USA) [38]. The laboratory personnel was blinded to the clinical status.

#### 2.1.3. Data Analysis

The data for continuous variables were expressed as mean value and standard deviation [mean ± SD] and data for nominal variables as count (or ratio) and percentage [n (%)]. The association between genotype distribution and disease was examined using the generalized odds ratio (OR_G_) [39,40]. In healthy controls, deviation of the genotype distribution from the Hardy–Weinberg equilibrium (HWE) was also tested. 

OR_G_ was calculated using ORGGASMA (http://biomath.med.uth.gr accessed on 30 August 2021) [39,40]. Statistical analysis was performed with SPSS version 26.0 for Windows (SPSS Inc., Chicago, IL, USA).

### 2.2. Meta-Analysis

#### 2.2.1. Identification and Eligibility of Relevant Studies

All of the studies published until December 2020 were identified by extended computer based search of PubMed database. The following search terms were used: (“Serpin family E member 1” or *SERPINE1* or “Plasminogen activator inhibitor-1” or PAI-1) AND (“diabetic nephropathy” OR “diabetic kidney disease” OR “end-stage renal disease”) AND (genetic or association or gene or polymorphism). We also retrieved articles from genome-wide association studies (GWASs) Catalog (https://www.ebi.ac.uk/gwas/ accessed on 1 December 2020). All of the references cited in the included studies were also scrutinized to identify additional published work. Meta-analyses of the included genes were also screened. Case reports, editorials, and review articles were also excluded. The search was restricted to articles in English.

Case–control studies that determined the distribution of genotypes harbored in *SERPINE1* gene in cases with DN and in either diseased controls with diabetes but without DN or in healthy controls were eligible for inclusion in the meta-analysis. The inclusion criteria of cases with DN and both diseased controls and healthy controls were the same as those of the present association study. Finally, genome linkage scans were excluded because they regard other study design.

#### 2.2.2. Data Extraction

From each article we extracted the following information: first author, year of publication, ethnicity, PubMed unique identifier, type of diabetes and phenotype. For cases and controls, we recorded their number, the selection criteria and the implementation of matching criteria. With regard to genotypic data, we extracted the full genotype counts or allele frequencies.

#### 2.2.3. Data Synthesis and Analysis

The association between genotype distribution and disease progression was examined using the generalized odds ratio (OR_G_) [39,40]. The threshold for meta-analysis was the presence of two studies per genetic variant. The pooled OR was estimated using the DerSimonian and Laird [41] random effects model. The associations are presented with ORs generalized for genotypic data with corresponding 95% confidence intervals (CIs). The between-study heterogeneity was tested with Cochran’s Q statistic (considered statistically significant at *p* < 0.10) [42] and we assessed its extent with the I^2^ statistic [43]. OR_G_ was calculated using a software for implementing the generalized odds ratio methodology for the analysis and meta-analysis of GAS (ORGGASMA) (http://biomath.med.uth.gr accessed on 30 August 2021).

For each study, we examined whether controls confronted with Hardy–Weinberg equilibrium (HWE) predicted genotypes using Fisher’s exact test. For studies providing only allele counts, we relied on the authors’ assessment of deviations from HWE. We also tested for small-study effects with the Egger test [44].

## 3. Results

### 3.1. Association Study

The cohort consisted of 190 cases (patients with T2DM and DN), 150 diseased controls (patients with T2DM without DN) and 238 healthy controls. All patients were Caucasians of Greek origin. The demographic and clinical characteristics are shown in Table 1. Among 190 cases with DN 11 were diagnosed with end-stage renal disease (ESRD). The patients were under treatment for chronic kidney disease, diabetes and hypertension including angiotensin-converting enzyme (ACE) inhibitors and angiotensin receptor blockers (ARB) as needed.

The genotype distributions of the five variants (rs2227667, rs2070682, rs1050813, rs2227690, rs2227692) in cases, diseased controls and healthy controls, and the respective OR_G_, are shown in Table 2. The healthy controls were conformed to HWE for all variants (*p* ≥ 0.05). There was no significant association in any polymorphism (*p* ≥ 0.05). We also examined the association between the five variants and disease progression taking into account all possible comparisons (Supplementary Tables S1–S5).

In addition, we examined the correlation between individual genotypes and clinical features (Table 3). Only rs2227692 variant was significantly correlated with creatinine levels (*p*-value = 0.037). Estimated glomerular filtration rate (eGFR) was also differed statistically significant between healthy controls, diseased controls and diabetic nephropathy cases (*p*-value < 0.001).

### 3.2. Meta-Analysis

**Figure 1:**
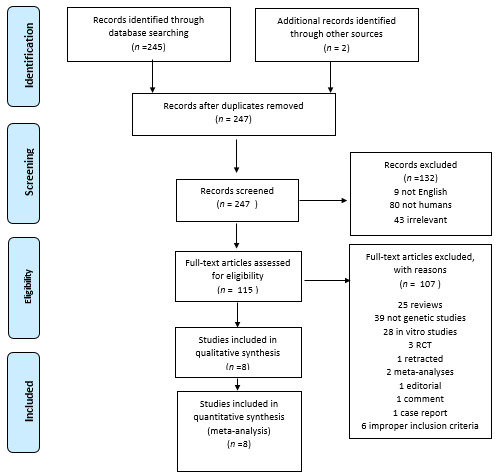
Flowchart of retrieved studies with reasons of exclusion.

**Figure 2:**
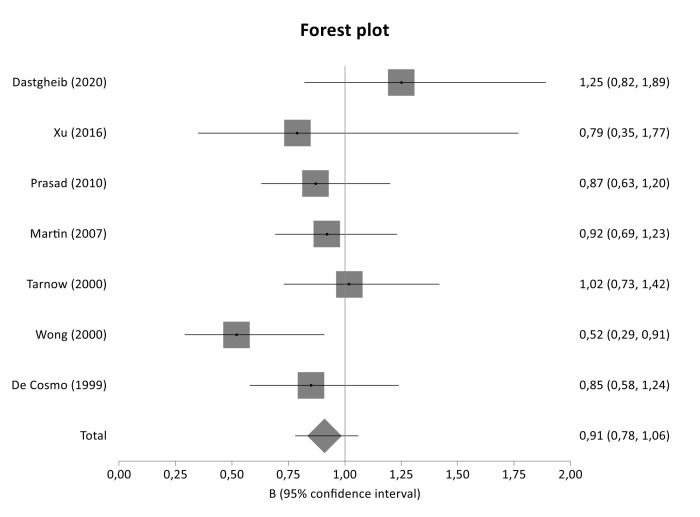
Forest plot of SERPINE1 4G/5G variant between diseased controls versus cases in main meta-analysis.

**Figure 3:**
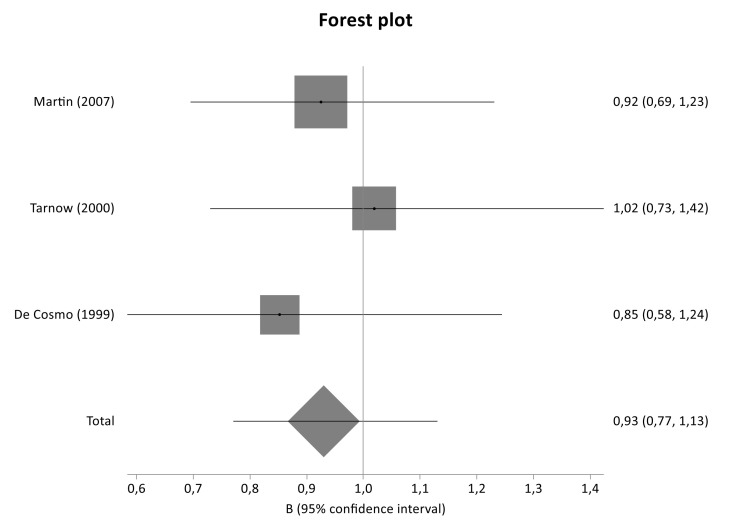
Forest plot of SERPINE1 4G/5G variant between diseased controls versus cases in T1DM subgroup meta-analysis.

**Figure 4:**
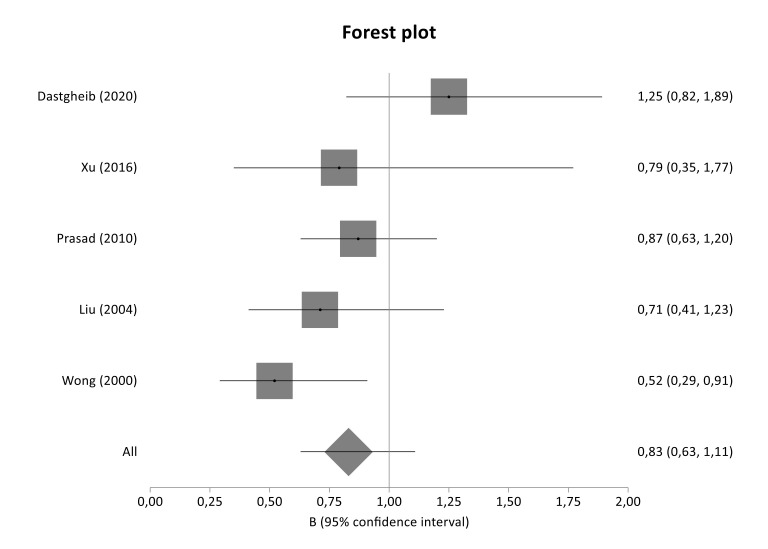
Forest plot of SERPINE1 4G/5G variant between diseased controls versus cases in T2DM subgroup meta-analysis.

The literature review identified 240 titles in PubMed that met the inclusion criteria. When an article provided data for different populations, each population was considered as a different study. Figure 1 presents a flowchart of retrieved and excluded articles with specifications of reasons for exclusion. The characteristics of each study are shown in Table 4. Across all available studies, only one polymorphism, 4G>5G (rs1799889), was examined in the context of genetic association studies regarding DN and so meta-analyzed. Statistical significance of rs1799889 was not reported in any analysis (Table 5). The studies comprised 1015 cases, 1001 diseased controls and 659 healthy controls and they were published between 1998 and 2016. Figure 2, Figure 3 and Figure 4 are forest plot representations of variant rs1799889.

## 4. Discussion

In an effort to provide the most comprehensive overview assessing for genetic variation in *SERPINE1* gene, we selected five tag SNPs for genotyping in a cohort of Greek origin and we also performed a meta-analysis that included all available genetic data regarding genetic variants of the aforementioned gene.

*SERPINE1* gene polymorphisms have been studied in various systematic reviews and meta-analyses regarding atherosclerotic diseases [45], risk of venous thromboembolism [46], stroke susceptibility [47] and diabetic nephropathy [32,38,48].

The present association study did not reveal statistical significance for any genotyped tag SNP located in *SERPINE1* gene. Similarly, the overall meta-analysis of 4G>5G variant (rs1799889) as well as the subgroup analyses based on diabetes type and ethnicity did not detect any significant association, indicating no implication of *SERPINE1* variants in the risk or development of the disease.

In agreement to the findings of the present genetic association study and meta-analysis, De Cosmo et al. (1999) found no association between *SERPINE1* 4G/5G polymorphism and DN in Europeans patients with T1DM [45]. Similarly, one more study in Caucasians with T1DM did not detect any significant association between twenty-one polymorphisms of *SERPINE1* and DN [29]. 

In contrast to our findings, Dastgheib et al. (2020) found that the PAI-1 4G5G polymorphism was associated with increased risk of DN and diabetic retinopathy (DR) risk [34]. Furthermore, Wong et al. (2000) concluded that *SERPINE1* 4G/4G is an independent factor for the development of DN in Chinese patients with T2DM and also exhibits a synergistic effect with the allele D of *ACE* gene on development of DN [27]. In addition, meta-analysis of Xue et al. (2014) reported a significant association between 4G/4G genotype and the risk of developing DN in overall analysis but also in both Asians and T2DM patients with DN [32]. Last but not least, one more meta-analysis showed that the *SERPINE1* 4G allele might be risk allele for DN susceptibility in the Chinese population [48].

Taking into account the discrepancy in findings derived from different study, it could be suggested that genetic variability in *SERPINE1* is influenced by the ethnicity and/or type of diabetes, as many studies conducted in Asians with T2DM detected significant association between genetic variants of *SERPINE1* and DN, whereas studies performed in Caucasians with T1DM did not detect any significant association between *SERPINE1* and DN. Our suggestion is in agreement with the conclusion of a recent meta-analysis of *SERPINE1* rs1799889 variant, in which Chen et al. (2021) concluded that 4G polymorphism could constitute a genetic synergistic factor in overall DM and DN populations, positively for individuals with Asian descent [49]. It is noteworthy to be mentioned that the association of rs1799889 variant was not revealed significant in diabetic retinopathy and cardiovascular risks [49].

Our study design has several strengths out of which clear case definition is one of them. Patients without persistent proteinuria were not considered as cases with DN, since not persistent proteinuria could be reversible and could lead to underestimation of the genetic effect. In addition, we included in the study, healthy controls without diabetes in order to discriminate any variant correlated with diabetes mellitus but not with DN per se. Moreover, the use of OR_G_ is a model-free approach, so it takes advantage of the full genotype distribution and provides a straightforward interpretation of genetic associations. On the other hand, the present study has certain limitations. The sample size of the present case-control study is relatively small raising the possibility of false positive and false negative results, an inherent though limitation of the majority of genetic association studies. Moreover, in the present meta-analysis only studies written in English language were included.

## 5. Conclusions

In conclusion, we investigated the role of *SERPINE1* gene polymorphisms in the context of a case-control study without statistical significant results for any variant. In an effort to further examine the role of *SERPINE1* gene variants in the risk of DN, we performed a systematic review and meta-analysis where 4G/5G polymorphism was also not revealed statistical significant. Further studies remain to verify the results of the present meta-analysis.

## Figures and Tables

**Table 1:** Clinical characteristics of the participants in the association study.

Parameters	Case-Control Study Population Groups (*n* = 578)					
	HC	DM	*p* Value	DM-DN	DM + DN	*p* Value
*N*	238	340	n.a.	150	190	n.a.
Gender [m; *n* (%)]	136 (42.9)	181 (57.1)	0.361	74 (47.7)	107 (54.3)	0.305
Age (years)	71 ± 9.2	68 ± 8.9	<.001	68 ± 9.1	69 ± 8.8	0.380
DM duration (years)	n.a.	16.3 ± 8.0	n.a.	15.7 ± 8.3	16.8 ± 7.8	0.203
HbA1c	n.d.	7.36 ± 1.32	n.a.	7.20 ± 1.34	7.47 ± 1.29	0.064
Insulin treatment (%)	n.d.	105 (32.3)	n.a.	50 (32.3)	55 (27.9)	0.412
Hypertension (%)	0	222 (63.4)	<.001	97 (63.0)	125 (63.8)	0.912
Cardiovascular disease (%)	0	110 (31.3)	<.001	41 (26.5)	69 (35.0)	0.105
Creatinine (mg/dL)	0.77 ± 0.15	1.46 ± 1.37	<.001	0.90 ± 0.18	1.85 ± 1.67	<0.001
Urea (mg/dL)	30 ± 7.9	59 ± 34	<.001	42 ± 13.6	71 ± 38.3	<0.001
UACR	36.7 ± 63.5	470 ± 856	0.382	43.9 ± 53.4	783 ± 1020	<0.001
Proteinuria (mg/dL)	136.6 ± 118.5	788 ± 1468	0.444	105 ± 80.0	1311 ± 1784	<0.001

**Table 2:** Genotype frequencies of the participants and results of the association study.

Variant	Genotype	DN			OR_G_ (95% CI)
		Healthy	Diseased Controls	Cases	
rs2227667	AA	110	72	100	0.85 (0.66, 1.08)
	GA	106	63	69	
	GG	18	14	14	
rs2070682	TT	81	51	74	0.97 (0.77, 1.23)
	TC	126	71	83	
	CC	30	25	32	
rs1050813	GG	158	113	129	0.92 (0.69, 1.23)
	AG	67	31	50	
	AA	8	5	6	
rs2227690	AA	153	93	132	0.87 (0.66, 1.15)
	GA	77	44	52	
	GG	6	7	5	
rs2227692	CC	184	119	153	0.88 (0.64, 1.23)
	CT	52	30	33	
	TT	2	1	4	

**Table 3:** Correlation of clinical features with individual genotypes.

Clinical Features	*p*-Value				
	rs2227667	rs2070682	rs1050813	rs2227690	rs2227692
**DM duration**	0.806	0.178	0.806	0.299	0.619
**HbA1c**	0.357	0.720	0.751	0.264	0.704
**Insulin**	0.522	0.223	0.927	0.871	0.712
**Hypertension**	0.644	0.144	0.849	0.392	0.826
**CVD**	0.644	0.334	0.945	0.464	0.788
**Creatinine**	0.500	0.199	0.491	0.480	0.037
**Urea**	0.498	0.674	0.138	0.290	0.687
**UACR**	0.306	0.688	0.328	0.833	0.609
**Proteinuria**	0.574	0.905	0.217	0.592	0.859
**eGFR**	0.195	0.970	0.088	0.310	0.609

**Table 4:** Demographic characteristics of the participants of the included studies in the meta-analysis.

SERPINE1 rs1799768	Dastgheib (2020) [34]	E. Asians	33520873	T2DM	DN	118	macr/ria	120	norm/ria without diabetic retinopathy				DC-C
	Xu (2016) [31]	E. Asians	26616527	T2DM	DN	33	macr/ria	44	norm/ria				DC-C, HT-DC-C, HT-C
	Prasad (2010) [30]	Asian Indians	20353610	T2DM	DN	196	CRI, serum Cr. ≥ 3.0 mg/dL	225	normal renal function and norm/ria, DM duration ≥ 10 yrs matched for age, ethnicity				DC-C
	Martin (2007) [29]	Caucasians	17263760	T1DM	DN	222	DM ≥ 10 yrs, pers. proteinuria, DR, no evidence of non-diabetic renal disease	361	DM > 15 yrs, pers. norm/ria, no anti-HT meds, background DR	86	non-diabetics		DC-C, HT-DC-C, HT-C
	Tarnow (2000) [26]	Caucasians	10809802	T1DM	DN	198	diabetic glomerulosclerosis, pers. macr/ria, retinopathy	192	pers. norm/ria age, gender, DM duration			No	DC-C
	Wong (2000) [27]	E. Asians	10652041	T2DM	DN	95	pers. micro/macroalbuminuriaor dialysis	46	pers. norm/ria, DM duration > 12 yrs matched for age, gender			No	DC-C, HT-DC-C, HT-C
	De Cosmo (1999) [25]	Caucasians	10495473	T1DM	DN	175	micro/macroalbuminuria, retinopathy	136	norm/ria, DM > 15 yrs	200	non-diabetics		DC-C, HT-DC-C, HT-C
	Kimura (1998) [24]	E. Asians	9844142	T2DM	DN	98	overt proteinuria, impaired renal function, DR or ESRD requiring dialysis		-	177	non-diabetics		HT-C

**Table 5:** Statistically significant results from meta-analysis of 4G/5G polymorphism.

**Diseased Controls versus Cases**										
Gene	Polymorphism	Rs number	N	Cases/Controls	RE OR_G_	LL OR_G_	UL OR_G_	I^2^	P_Q_	P_E_
SERPINE1	c.-821_-820insG (4G>5G)	rs1799768	7	1035/1121	0.91	0.78	1.06	6.76	0.34	0.13
SERPINE1	All in HWE		4	626/764	0.88	0.74	1.06	0	0.98	0.17
T1DM/Caucasians										
SERPINE1	4G>5G	rs1799768	3	594/688	0.93	0.77	1.13	0	0.78	0.08
T2DM/Asians										
SERPINE1	4G>5G	rs1799768	4	518/503	0.85	0.60	1.21	51.08	0.11	0.15
SERPINE1	4G>5G	rs1799768	2	400/383	0.86	0.64	1.16	0	0.83	0.12
**Healthy Controls versus Diseased Controls versus Cases**										
SERPINE1	c.-821_-820insG (4G>5G)	rs1799768	4		0.9	0.76	1.05	14.57	0.32	0
T1DM/Caucasians										
SERPINE1			2		0.96	0.81	1.14	0	0.83	-
T2DM/Asians										
SERPINE1			2		0.74	0.53	1.04			-
**Healthy Controls versus Cases**										
SERPINE1	c.-821_-820insG (4G>5G)	rs1799768	5	622/659	0.92	0.74	1.13	9.02	0.36	0.07
T1DM/Caucasians										
SERPINE1	c.-821_-820insG (4G>5G)	rs1799768	2		0.96	0.73	1.25	0	0.70	-
T2DM/Asians										
SERPINE1	c.-821_-820insG (4G>5G)	rs1799768	3		0.83	0.55	1.27	50.87	0.13	-

## Data Availability

The datasets used and/or analysed during the current study are available from the corresponding author on reasonable request.

## Supplementary Materials

The following are available online at https://www.mdpi.com/article/10.3390/genes12121887/s1, Table S1: Genotype frequencies of the participants and results of the association study between healthy controls versus diabetics (with and without diabetic nephropathy), Table S2: Genotype frequencies of the participants and results of the association study between cases versus controls (with and without diabetes), Table S3: Genotype frequencies of the participants and results of the association study between healthy and diseased controls, Table S4: Genotype frequencies of the participants and results of the association study between healthy controls versus cases with diabetic nephropathy, Table S5: Genotype frequencies of the participants and results of the association study between diseased controls versus cases with diabetic nephropathy.

## Abbreviations

DN	diabetic nephropathy;
GWASs	Genome-Wide Association Studies;
GWLS	Genome-Wide Linkage Studies;
HWE	Hardy–Weinberg equilibrium;
MAF	Minor Allele Frequency;
OR_G_	Generalized Odds Ratio;
PAI-1	Plasminogen activator inhibitor-1;
serpin	serine proteinase inhibitor;
*SERPINE1*	serpin family E member 1;
SNPs	Single-Nucleotide Polymorphisms;
T1DM	type 1 diabetes mellitus;
T2DM	type 2 diabetes mellitus;
tPA	tissue plasminogen activator;
uPA	urokinase.
