# Supplementary material for: Serpin Family E Member 1 Tag Single-Nucleotide Polymorphisms in Patients with Diabetic Nephropathy: An Association Study and Meta-Analysis Using a Genetic Model-Free Approach
Source: Genes (Basel). 2021 Nov 25;12(12):1887. doi: 10.3390/genes12121887 (PMC8701119; doi:10.3390/genes12121887)
Supplement: Supplementary file 1 [file genes-12-01887-s001.zip › genes-1443309-supplementary.pdf]

Table S1: Genotype frequencies of the participants and results of the association study between healthy controls versus diabetics (with and without diabetic nephropathy).

Variant	Genotype	DM		ORG (95% CI)
		Healthy	DM (Diseased Controls and Cases)	
rs2227667	AA	110	172	0.87 (0.64, 1.17)
	GA	106	132	
	GG	18	28	
rs2070682	TT	81	125	1.01 (0.76, 1.34)
	TC	126	154	
	CC	30	57	
rs1050813	GG	158	242	0.81 (0.57, 1.15)
	AG	67	81	
	AA	8	11	
rs2227690	AA	153	225	0.91 (0.65, 1.27)
	GA	77	96	
	GG	6	12	
rs2227692	CC	184	272	0.86 (0.58, 1.28)
	CT	52	63	
	TT	2	5	

Table S2: Genotype frequencies of the participants and results of the association study between cases versus controls (with and without diabetes).

Variant	Genotype	No DN (Healthy & Diseased Controls)	DN Cases	ORG (95% CI)
rs2227667	AA	182	100	0.78 (0.57, 1.08)
	GA	169	69	
	GG	32	14	
rs2070682	TT	132	74	0.93 (0.68, 1.26)
	TC	197	83	
	CC	55	32	
rs1050813	GG	271	129	1.05 (0.73, 1.52)
	AG	98	50	
	AA	13	6	
rs2227690	AA	246	132	0.80 (0.55, 1.15)
	GA	121	52	
	GG	13	5	
rs2227692	CC	303	153	0.88 (0.57, 1.35)
	CT	82	33	
	TT	3	4	

Table S3: Genotype frequencies of the participants and results of the association study between healthy and diseased controls.

Variant	Genotype	Healthy	Diseased controls	OR$_G$ (95% CI)
rs2227667	AA	110	72	0.99 (0.68, 1.44)
	GA	106	63	
	GG	18	14	
rs2070682	TT	81	51	1.08 (0.75, 1.56)
	TC	126	71	
	CC	30	25	
rs1050813	GG	158	113	0.69 (0.44, 1.08)
	AG	67	31	
	AA	8	5	
rs2227690	AA	153	93	1.04 (0.69, 1.59)
	GA	77	44	
	GG	6	7	
rs2227692	CC	184	119	0.89 (0.54, 1.45)
	CT	52	30	
	TT	2	1	

Table S4: Genotype frequencies of the participants and results of the association study between healthy controls versus cases with diabetic nephropathy.

Variant	Genotype	DM_DN		OR$_G$ (95% CI)
		Healthy	DN Cases	
rs2227667	AA	110	100	0.78 (0.54, 1.11)
	GA	106	69	
	GG	18	14	
rs2070682	TT	81	74	0.95 (0.68, 1.33)
	TC	126	83	
	CC	30	32	
rs1050813	GG	158	129	0.92 (0.61, 1.37)
	AG	67	50	
	AA	8	6	
rs2227690	AA	153	132	0.81 (0.54, 1.20)
	GA	77	52	
	GG	6	5	
rs2227692	CC	184	153	0.84 (0.53, 1.34)
	CT	52	33	
	TT	2	4	

Table S5: Genotype frequencies of the participants and results of the association study between diseased controls versus cases with diabetic nephropathy.

Variant	Genotype	DM_DN		OR_G (95% CI)
		Diseased Controls	DN Cases	
rs2227667	AA	72	100	0.79 (0.53, 1.17)
	GA	63	69	
	GG	14	14	
rs2070682	TT	51	74	0.89 (0.61, 1.28)
	TC	71	83	
	CC	25	32	
rs1050813	GG	113	129	1.33 (0.83, 2.14)
	AG	31	50	
	AA	5	6	
rs2227690	AA	93	132	0.78 (0.50, 1.21)
	GA	44	52	
	GG	7	5	
rs2227692	CC	119	153	0.95 (0.56, 1.60)
	CT	30	33	
	TT	1	4	
